# Invincibility threatens vaccination intentions during a pandemic

**DOI:** 10.1371/journal.pone.0258432

**Published:** 2021-10-27

**Authors:** James M. Leonhardt, Garret Ridinger, Yu Rong, Amir Talaei-Khoe

**Affiliations:** 1 Department of Marketing, University of Nevada, Reno, Nevada, United States of America; 2 Department of Management, University of Nevada, Reno, Nevada, United States of America; 3 Department of Information Systems, University of Nevada, Reno, Nevada, United States of America; UCLA Fielding School of Public Health, UNITED STATES

## Abstract

Some people feel they are invincible to the novel coronavirus SARS-CoV-2 (COVID-19). They believe that being infected with COVID-19 would not be a serious threat to their health. While these people may or may not be correct in their personal risk assessment, we find that such perceived invincibility may undermine community efforts to achieve herd immunity. Multi-level analysis of survey respondents across 51 countries finds that perceived invincibility from COVID-19 is negatively associated with believing there is a need to prevent the spread of COVID-19 in one’s community (*n* = 218,956) and one’s willingness to inoculate against the disease (*n* = 71,148). These effects are most pronounced among individuals from countries lower in cultural collectivism (e.g., USA, UK, Canada) and highlight the need to consider the interplay of individual and cultural factors in our efforts to understand, predict, and promote preventative health behavior during a pandemic.

## Introduction

Despite scientific evidence to the contrary, some people feel they are invincible to life-threatening diseases. In the context of the novel coronavirus SARS-CoV-2 (COVID-19) pandemic, such feelings of invincibility pose challenges for suppressing the disease and achieving herd immunity at global scale [1–3]. Prosocial concern and action are needed to help suppress the disease—people need to be concerned about taking preventive measures (e.g., mask wearing) to help prevent the spread of the disease and they need to be willing to inoculate against the disease to help communities achieve herd immunity [4–6]. However, prosocial actions aimed at curbing the spread of COVID-19 may depend on the extent that people feel invincible to COVID-19-related illness.

The present research examines whether believing that COVID-19 is not a serious threat to one’s health (i.e., perceived invincibility) negatively affects one’s belief in the importance of taking preventative action to suppress the spread of the disease. Using largescale cross-cultural survey data across 51 countries, we find evidence that perceived invincibility from COVID-19 negatively affects the extent that people are concerned about preventing the spread of the disease in their community (*n* = 218,956) and their willingness to inoculate against the disease (*n* = 71,148). Moreover, building on cultural dimensions theory [7, 8], we find that these relationships are most pronounced among people from countries lower in cultural collectivism (e.g., USA, UK, Canada). These findings contribute to our understanding of the interplay between individual and cultural-level factors in affecting prosocial beliefs and behaviors, which are crucial for suppressing a highly contagious disease, such as COVID-19.

Prior research has considered a number of individual and cultural-level factors that contribute to prosocial beliefs and behaviors related to COVID-19 [4, 9–17]. We contribute to this effort by identifying perceived invincibility as an individual-level difference factor that could threaten collective efforts to suppress the pandemic at global scale. Moreover, given that we find modulation by cultural collectivism, the threat of perceived invincibility may be especially pronounced in less collectivistic cultures where there is greater emphasis on individual freedoms and autonomy [8]. Especially in less collectivistic cultures, we suggest that health and public policy communications encourage a collectivistic mindset by stressing our interdependence and the idea that our personal wellbeing and that of others is dependent on collective concern and action.

## Background and hypothesis development

### Perceived invincibility

Invincibility refers to the unrealistic belief of invulnerability or success in overcoming risky prospects [18]. Brown [19] defines invincibility as an individual’s unrealistic belief that “s/he will be successful in any endeavor, including high-risk ones” [19 p2]; and Wickman, Anderson, and Grenberg [20] characterize invincibility as the belief that “it won’t happen to me” [20 p461]. Perceived invincibility encourages feelings of self-assuredness and cockiness [18] and is thought to arise from egocentric thinking, which can lead to feelings of uniqueness, exceptionalism, and the sense that one is not subject to the same risks or laws governing the lives of others [21, 22]. In turn, perceived invincibility promotes risk taking, including health-related risk-taking [19, 20, 23]. Perceived invincibility is associated with less precaution when driving a car and having sex [23–26], lower willingness to screen for testicular cancer [27], excessive alcohol consumption [28], and less receptivity to public-health communications on the risks of overdose among heroin users [24].

In addition to threatening personal health, we ask whether perceived invincibility might also threaten the wellbeing of others, especially during a pandemic? The suboptimal response to COVID-19 by some developed countries has been attributed to feelings of invincibility, as Kavanagh, Pare and Pontus [29] note, “The belief in invincibility and that a pandemic could never reach the United States (U.S.) led to a depletion of personal protective equipment (PPE), including N-95 masks in the U.S. stockpile.” [29 p2]. Moreover, U.S. President Donald Trump seemingly promoted feelings of invincibility through his messaging on Twitter, e.g., “Don’t be afraid of COVID. Don’t let it dominate your life.” [30]. Such messaging is not unique to the recent political landscape, however, as it can be seen during the Civil War in the United States with the Confederate’s emphasis on a culture of invincibility [31]. And while there may be advantages to invincibility (e.g., overcoming economic hardships), it could result in a suboptimal response during a pandemic when prosocial beliefs and behaviors are needed to collectively suppress a highly contagious disease.

### Prosocial beliefs and behavior

To eradicate a pandemic such as COVID-19, prosocial beliefs and behaviors are needed on the behalf of all community members, despite feelings of personal invincibility [3, 5, 6, 32]. To suppress COVID-19, individuals need to be concerned about the spread of the disease in their community, which should encourage preventative behaviors to lessen its spread, e.g., handwashing, social distancing, and mask wearing [1, 2, 33]. Willingness to inoculate is also needed such that a sufficient proportion of the population becomes resistant to the disease, which significantly reduces the rate of disease transmission [1]. Moreover, achieving herd immunity is especially important for protecting those most susceptible to the disease and those for whom inoculation is less effective at increasing immunity [33, 34].

Vaccines provide direct benefits to individuals and indirect benefits to one’s community. Inoculated individuals are at a much lower risk of severe illness or death from COVID-19, and they are much less likely to spread the disease to others [1, 4]. However, there are also personal costs associated with vaccination [35, 36], which could increase vaccination hesitancy in the absence of prosocial concern about reducing disease transmission [5, 32, 37]. Recent research suggests that vaccination is a moral obligation or social contract, such that to suppress a disease through inoculation, prosocial motivation is essential in addition to self-interested factors [5, 32]. In the U.S., prosocial concern about infecting others was positively associated with influenza vaccinations [6]; and in Israel, prosocial, rather than self-interested, motives contributed to polio vaccine (OPV) uptake [37]. In turn, prosocial concern, defined herein as one’s concern about taking action to reduce COVID-19 transmission in one’s community, may play a critical role in suppressing the disease.

Feeling personally invincible from the health threats posed by a highly contagious disease, such as COVID-19, might lessen the extent that individuals are concerned about and take action to suppress the spread of the disease in their community. Individual differences and demographic factors may contribute toward differential feelings of personal susceptibility to serious illness from the disease and could foster a sense of invincibility to the negative health ramifications of the disease [3, 4, 10, 13, 16, 17]. Older members of the population and those with chronic illness are at higher risk of life-threatening complications from COVID-19, while young and healthy members of the population are at a lower risk, and many are asymptomatic [34, 38]. As a result, younger people may feel less vulnerable to COVID-19 and may be less willing to engage in preventative health behaviors [13, 39].

Gender can also affect invincibility perceptions, with males typically scoring higher on perceived invincibility [27, 40, 41]. Recent research on COVID-19 finds that compared to females, males are more likely to believe they will be unaffected by the disease, and they are more hesitant to engage in preventative health behaviors, such as mask wearing, hand washing, social distancing, and encouraging others to take health precautions [13, 15, 17, 39, 42]. Personality traits such sensation seeking, impulsivity, and risk preference are also associated with an individual’s propensity to take risks [43–45] and may enhance the extent that individuals feel invincible from COVID-19. Invincibility is also known to be associated with egocentric thinking and feelings of uniqueness and independence from others [21, 22], which can lessen cooperative and prosocial behavior [46, 47].

In the context of COVID-19, research suggests that perceived invincibility may be negatively associated with one’s concern about preventing the spread of the disease and one’s willingness to inoculate against the disease. Formally, we predict: Perceived invincibility from COVID-19 illness will negatively relate to the belief that one should take action to prevent the spread of COVID-19 in their community (i.e., prosocial concern; H1a) and one’s willingness to inoculate against the disease (i.e., vaccination intention; H1b).

### Cultural collectivism

While invincibility is expected to negatively relate to prosocial concern about preventive behaviors and willingness to inoculate against COVID-19, these relationships may depend on an individual’s cultural orientation. Collectivism is a prominent influence on one’s cultural orientation and varies considerably across countries [8]. Collectivism affects one’s self-perception in relation to others [46, 48]. With a collectivistic cultural orientation, individuals perceive themselves as interdependent and reliant on the wellbeing of others, in addition to themselves. The lack of a collectivistic orientation, on the other hand, results in individuals perceiving themselves as independent, autonomous, and less responsible for the needs or wellbeing of others [46, 49].

As a result of feeling greater interdependence with and concern for the wellbeing of community members, cultural collectivism may promote prosocial concern and vaccination intentions, despite personal feelings of invincibility from COVID-19. Recent research finds a positive relationship between collectivism and empathetic concern for those afflicted by COVID-19 [50], and empathy has been shown to promote physical distancing and the wearing of face masks during the COVID-19 pandemic [11]. Collectivism was also found to relate positively to social distancing intentions during the pandemic [51]. Fincher et al. [52] anticipated such cross-cultural differences in prosocial beliefs and behaviors in response to the pandemic by suggesting that the often-higher prevalence of pathogens in collectivistic cultures has encouraged preventive behaviors and prosocial concern in response to deadly outbreaks.

In the context of COVID-19, research suggests that perceived invincibility’s threat to taking collective action to suppress the pandemic may be less pronounced in collectivistic cultures where an emphasis is placed on collective rather than personal needs. Formally, we predict: Cultural collectivism will moderate the relationship between perceived invincibility and prosocial concern and vaccination intention, such that cultural collectivism will attenuate the otherwise negative relationship between perceived invincibility and the belief that one should take action to prevent the spread of COVID-19 in their community (H2a) and one’s willingness to inoculate against the disease (H2b).

## Method

Our hypotheses were tested using a largescale cross-cultural survey conducted during the COVID-19 pandemic. Specifically, we assessed the relationship between the perceived threat of COVID-19 to one’s health (i.e., perceived invincibility) and one’s belief in the importance of taking action to prevent the spread of COVID-19 in one’s community (i.e., prosocial concern; H1a) and whether this relationship is modulated by cultural collectivism (H2a). Similarly, we assessed the relationship between perceived invincibility and one’s willingness to inoculate against COVID-19 (i.e., vaccination intention; H1b) and whether this relationship is also modulated by cultural collectivism (H2b).

### Individual-level COVID-19 beliefs

Individual-level data was obtained from the Beliefs, Behaviors, and Norms Survey (BBNS) administered by the Massachusetts Institute of Technology and Facebook’s Data for Good [53, 54]. Survey participants were recruited by Facebook using targeted display ads to generate representative country-level samples (Fig A in S1 File). The survey was ongoing during the pandemic, and data used herein was collected from July to November 2020. See S1 File for details on the survey instrument and methodology. Academic and non-profit researchers may request access to the survey dataset by completing the Facebook Data Use Agreement at https://dataforgood.fb.com/docs/preventive-health-survey-request-for-data-access/.

From the BBNS, we sourced the following measures to assess our hypotheses. Perceived invincibility to the health risks posed by COVID-19 infection was measured by asking participants, “How serious would it be if you became infected with COVID-19?” (1 = *Not at all serious*, 2 = *Somewhat serious*, 3 = *Very serious*). Prosocial concern about taking actions to prevent the spread of COVID-19 was measured by asking participants, “How important is it for you to take actions to prevent the spread of COVID-19 in your community?” (1 = *Not important at all*, 2 = *Slightly important*, 3 = *Moderately important*, 4 = *Very important*, 5 = *Extremely important*). Vaccination intentions were measured by asking participants, “If a vaccine against COVID-19 infection is available in the market, would you take it?” (1 = *No*, *definitely not*, 2 = *Probably not*, 3 = *Unsure*, 4 = *Probably*, 5 = *Yes*, *definitely*).

Several control variables were also included. Perceived personal health was measured by asking participants, “In general, how would you rate your overall health?” (1 = *Poor*, 2 = *Fair*, 3 = *Good*, 4 = *Very good*, 5 = *Excellent*). Demographic measures included age (1 = *Under 20*, 2 = *20–30*, 3 = *31–40*, 4 = *41–50*, 5 = *51–60*, 6 = *61–70*, 7 = *71–80*, 8 = *Over 80*), sex (0 = *Female*, 1 = *Male*), and education (1 = *Less than primary school*, 2 = *Primary school*, 3 = *Secondary school*, 4 = *College/university*, 5 = *Graduate school*).

### Country-level cultural collectivism

Cultural collectivism was estimated using country-level data from four prior studies [7, 55–57]. Following Fincher et al. [52] and Webster et al. [58], the four measures were standardized (z-score), and the Hofstede [7] and Suh et al. [57] measures we reverse-scored so that higher scores represented higher cultural collectivism [58]. This procedure resulted in cultural collectivism scores for 51 countries that corresponded with the BBNS individual-level data. The four measures of cultural collectivism were significantly correlated (mean bivariate correlation = .79, α = .95) and were averaged to create an index measure, which was linearly transformed to have a mean of 50.37 (*SD* = 17.44) [58, 59]. S1 File provides additional methodological details, and the data are available from the OSF repository at https://osf.io/qwn9f/?view_only=4349d3e12ade40b99b10053cf4f8fdf8.

## Results and discussion

### Prosocial concern

Linear mixed models were estimated to account for the nested nature of the individual-level and country-level measures [60, 61]. The first model assessed the relationship between perceived invincibility and prosocial concern (H1a) and the interaction between cultural collectivism and perceived invincibility on prosocial concern (H2a). The model included prosocial concern as the dependent measure (*n* = 218, 956) and perceived invincibility and cultural collectivism (*n* = 51) as predictor variables, along with the control variables. For the linear mixed models, the specific functional form utilized were random intercept models. This modeling approach includes an intercept at the individual level but allows for random-effect intercepts at the country level. The models were estimated using restricted maximum likelihood [62]. At the individual-level, fixed effects included perceived invincibility, cultural collectivism, and their interaction, along with the control variables. To control for country variation, random intercepts were estimated at the country level. Notably, regression results for prosocial concern and vaccine intentions were similar when including health, age, and education as categorical variables instead of continuous. Additional analysis, described below, also assessed model robustness across age cohorts and gender. See S1 File for descriptive statistics and alternative model specifications to which the results are generally robust.

Model results are shown in Table 1. In column (1), perceived invincibility was treated as a continuous variable predicting prosocial concern. There were significant effects of age (*b* = .018, *z* = 15.25, *p* < .01), education (*b* = .060, *z* = 26.64, *p* < .01), and sex (*b* = -.141, *z* = -39.72, *p* < .01) on prosocial concern, indicating that older participants, more educated participants, and female participants were more concerned with taking action to prevent the spread of COVID-19 in their community. There was a significant main effect of cultural collectivism on prosocial concern (*b* = -.010, z = -5.85, *p* < .01); and, as expected, there was a significant main effect of perceived invincibility on prosocial concern (*b* = -.707, z = -90.37, *p* < .01). This result supports H1a and suggests that higher perceived invincibility from COVID-19 infection results in lower prosocial concern. However, qualifying this relationship was the posited interaction between perceived invincibility and cultural collectivism (*b* = .007, z = 50.00, *p* < .01).

**Table 1:** Linear mixed model results for the effects of perceived invincibility and cultural collectivism on prosocial concern.

	(1)					(2)				
	Est.	SE	p-Value	95% CI	Est.	SE	p-Value	95% CI
			*LL*	*UL*			*LL*	*UL*
*Fixed Effects*										
Constant	5.03	0.09	<0.001	4.85	5.22	4.20	0.09	<0.001	4.02	4.39
Perceived Invincibility (PI)	-0.71	0.01	<0.001	-0.72	-0.69					
Cultural Collectivism (CC)	-0.01	0.00	<0.001	-0.01	-0.01	-0.00	0.00	0.614	-0.00	0.00
PI x CC	0.01	0.00	<0.001	0.01	0.01					
High Perceived Invincibility (High PI)						-1.63	0.02	<0.001	-1.66	-1.60
Medium Perceived Invincibility (Medium PI)						-0.38	0.01	<0.001	-0.40	-0.36
High PI x CC						0.02	0.00	<0.001	0.02	0.02
Medium PI x CC						0.00	0.00	<0.001	0.00	0.00
Age	0.02	0.00	<0.001	0.01	0.02	0.02	0.00	<0.001	0.02	0.02
Sex (male)	-0.14	0.00	<0.001	-0.15	-0.13	-0.14	0.00	<0.001	-0.14	-0.13
Education	0.06	0.00	<0.001	0.06	0.07	0.06	0.00	<0.001	0.05	0.06
Health	0.06	0.00	<0.001	0.05	0.06	0.06	0.00	<0.001	0.06	0.07
*Random Effects*										
Country	0.04	0.01		0.03	0.06	0.04	0.01		0.03	0.06
Residual	0.62	0.00		0.61	0.62	0.61	0.00		0.61	0.61
										
Wald Chi Sq. (9) 22860.09						25423.72				
Prob > Chi Sq. 0.000						0.000				
# of Observations 218,956						218,956				

**Fig 1:**
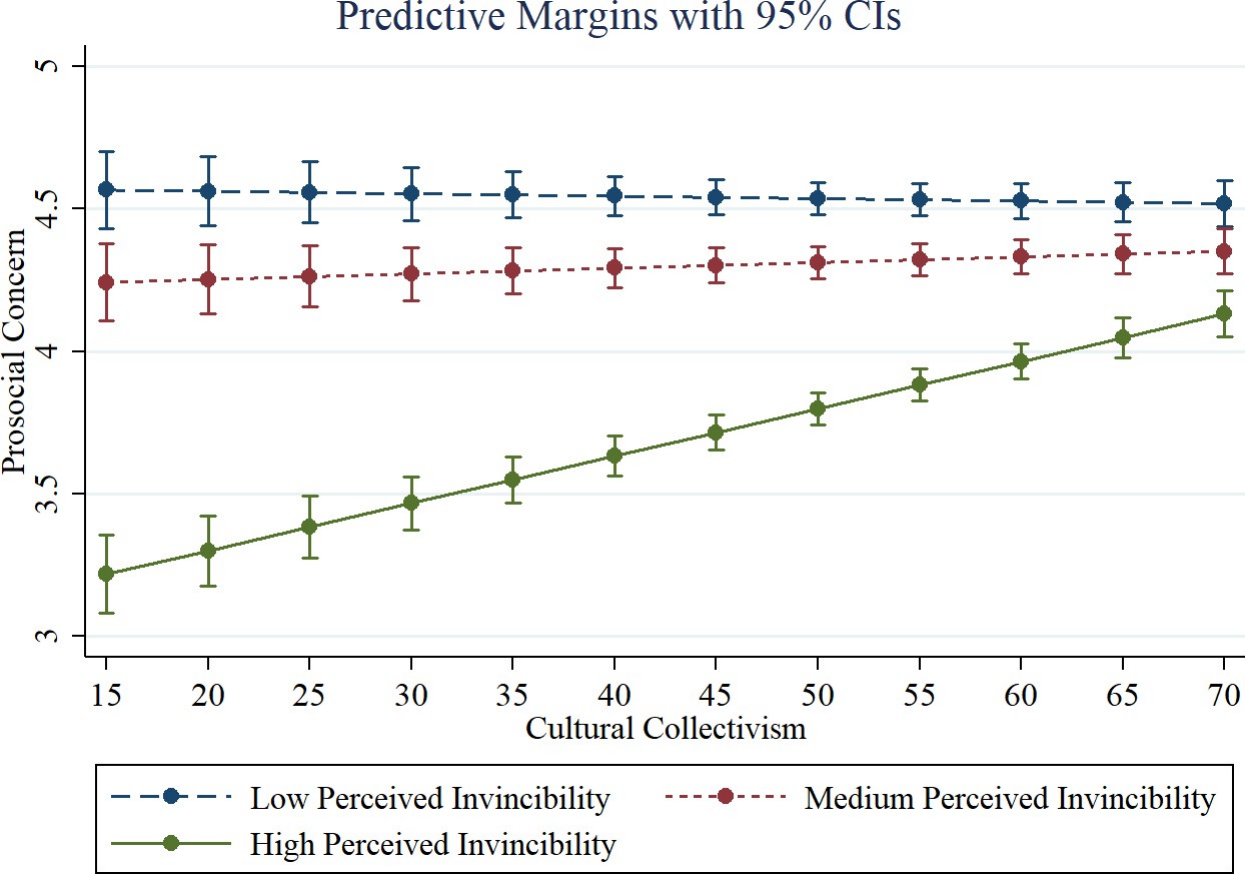
Predicted prosocial concern by perceived invincibility and cultural collectivism. Fig 1 uses linear mixed methods results from Table 1 Column (2).

In Table 1 column (2), perceived invincibility was specified as a categorical variable on predicted prosocial concern. Those who indicated medium (*Somewhat serious*) and high (*Not at all serious*) perceived invincibility were significantly less concerned about taking action to prevent the spread of COVID-19 in their community compared to those with low (*Very serious*) perceived invincibility (*b* = —.379, *z* = -33.75, *p* < .01; *b* = -1.63, *z* = -97.66, *p* < .01). Additionally, this effect on prosocial concern was larger for high compared to medium perceived invincibility (*Chi-square*(1) = 6035.10, *p* < .01). The main effect of cultural collectivism on prosocial concern captures the effect of cultural collectivism among those with low perceived invincibility (the omitted category) and was non-significant (*b* = -.001, *z* = -0.50, *p* = .61). The interaction between cultural collectivism and medium perceived invincibility was positive and significant (*b* = .003, *z* = 13.58, *p* < .01). Additionally, the interaction between high perceived invincibility and cultural collectivism was positive and significant (*b* = .017, *z* = 55.04, *p* < .01). Fig 1 illustrates these findings by plotting the interaction between cultural collectivism and the categories of perceived invincibility on predicted prosocial concern; while perceived invincibility has an overall negative effect on prosocial concern, the effect is particularly pronounced among participants in cultures with low cultural collectivism.

### Vaccination intention

As in the previous analysis, a linear mixed model was estimated to test the proposed relationship between perceived invincibility on vaccination intention (H1b), and the interaction between cultural collectivism and perceived invincibility on vaccination intention (H2b). The model included vaccination intention as the dependent variable (*n* = 71,148), perceived invincibility and cultural collectivism (*n* = 51) as predictor variables, and the same control variables. Fixed effects included perceived invincibility, cultural collectivism, and their interaction, along with the control variables and, as in the previous analysis, to control for country variation, random intercepts were estimated at the country level. See S1 File for descriptive statistics and correlations.

Model results are shown in Table 2. In column (1), perceived invincibility was specified as a continuous variable predicting vaccine intention. There were significant effects of education (*b* = .093, *z* = 15.49, *p* < .01) and sex (*b* = .236, *z* = 25.02, *p* < .01) on vaccination intention, indicating that more educated participants and male participants were more willing to inoculate themselves against COVID-19. The effect of age on vaccine intention was significant (*b* = .001, *z* = 2.22, *p* < .05), indicating that older participants were more willing to be vaccinated. There was a non-significant main effect of cultural collectivism on vaccination intention (*b* = -.006, *z* = -1.86, *p* = .062). As expected, there was a significant main effect of perceived invincibility on vaccination intention (*b* = -.602, *z* = -29.08, *p* < .01). This result supports H1b and suggests that higher perceived invincibility from COVID-19 infection results in lower willingness to inoculate against COVID-19. However, qualifying this relationship was the positive interaction between invincibility and cultural collectivism (*b* = .005, *z* = 12.08, *p* < .01). As expected, cultural collectivism weakened the otherwise robust negative relationship between perceived invincibility and vaccination intention.

**Table 2:** Linear mixed model results for the effects of perceived invincibility and cultural collectivism on vaccination intention.

	(1)					(2)				
	Est.	SE	p-Value	95% CI	Est.	SE	p-Value	95% CI
				*LL*	*UL*				*LL*	*UL*
*Fixed Effects*										
Constant	4.32	0.19	<0.001	3.94	4.69	3.57	0.19	<0.001	3.20	3.94
Perceived Invincibility (PI)	-0.60	0.02	<0.001	-0.64	-0.56					
Cultural Collectivism (CC)	-0.01	0.00	0.06	-0.01	0.00	0.00	0.00	0.759	-0.05	0.01
PI x CC	0.01	0.00	<0.001	0.00	0.01					
High Perceived Invincibility (High PI)						-1.43	0.04	<0.001	-1.52	-1.35
Medium Perceived Invincibility (Medium PI)						-0.22	0.03	<0.001	-0.28	-.16
High PI x CC						0.01	0.00	<0.001	0.01	0.01
Medium PI x CC						0.00	0.00	0.394	-0.00	0.00
Age	0.01	0.0	0.026	0.00	0.01	0.01	0.00	0.008	0.00	0.02
Sex (male)	0.24	0.01	<0.001	0.22	0.25	0.24	0.01	<0.001	0.22	0.26
Education	0.09	0.01	<0.001	0.08	0.11	0.08	0.01	<0.001	0.07	0.10
Health	-0.03	0.01	<0.001	-0.03	-0.02	-0.02	0.00	<0.001	-0.03	-0.01
*Random Effects*										
Country	0.12	0.03		0.08	0.17	0.12	0.02		0.08	0.17
Residual	1.40	0.01		1.39	1.42	1.39	0.01		1.38	1.41
										
Wald Chi Sq. (9) 3927.12						4636.00				
Prob > Chi Sq. 0.000						0.000				
# of Observations 71,148						71,148				

**Fig 2:**
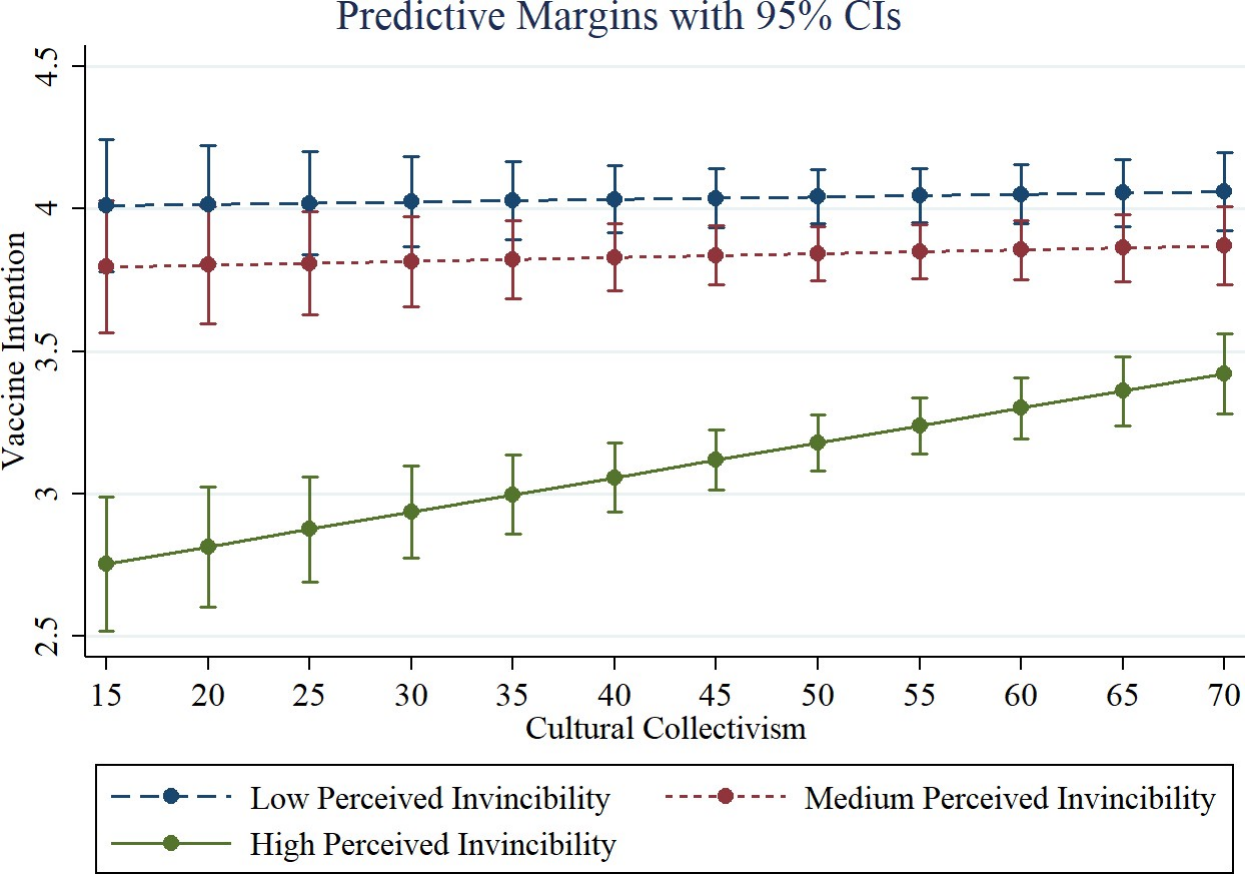
Predicted vaccine intention by perceived invincibility and cultural collectivism. Fig 2 uses linear mixed methods results from Table 2 Column (2).

In Table 2 column (2), perceived invincibility was specified as a categorical variable predicting vaccination intention. Those who indicated medium (*Somewhat serious*) and high (*Not at all serious*) perceived invincibility were significantly less willing to get the vaccine compared to those with low (*Very serious*) perceived invincibility (*b* = -.222, *z* = -7.48, *p* < .01; *b* = -1.43, *z* = -32.50, *p* < .01). Additionally, this effect on vaccination intention was larger for high compared to medium perceived invincibility (*Chi-square*(1) = 819.87, *p* < .01). The main effect of cultural collectivism on vaccination intention, which captures the effect of cultural collectivism among those with low perceived invincibility (the omitted category), was non-significant (*b* = .000, *z* = 0.31, *p* = .759). The interaction between cultural collectivism and medium perceived invincibility was also non-significant (*b* = .000, *z* = 0.85, *p* = .394). However, the interaction between high perceived invincibility and cultural collectivism was positive and significant (*b* = .011, *z* = 13.58, *p* < 0.01). Fig 2 illustrates these findings by plotting the interaction between cultural collectivism and the categories of perceived invincibility on predicted vaccination intention. While invincibility has an overall negative effect on vaccination intention, the effect is particularly pronounced among participants in cultures with low cultural collectivism.

### Additional analysis

Additional analysis is warranted given that perceptions of invincibility have been found to be correlated with both age and gender [13, 15, 17, 39, 42]. While our prior analyses controlled for age and gender, it is possible that the effect of perceived invincibility on both prosocial concern and vaccination intention differs by these demographic characteristics. We conducted a series of regression analyses to verify whether the effects found in our prior analysis are robust across age and sex.

For each age cohort, we ran separate regressions predicting the dependent variables: prosocial concern and vaccination intention. The independent variables included perceived invincibility, cultural collectivism, and their interaction, along with the same control variables as in our prior analysis. Notably, due to lower sample sizes for the age groups “under 20” and “over 80” compared to the other age groups, caution is warranted in drawing conclusions about the estimated effects for those age groups compared to the other age cohorts. See S1 File for additional details.

For prosocial concern (Tables H and I in S1 File), the coefficients for perceived invincibility were negative and significant across all age cohorts. The coefficients for cultural collectivism were negative and significant across all ages except those under the age of 20. The interaction effects between perceived invincibility and cultural collectivism were significant and positive for all ages except those over the age of 80. For vaccination intention (Tables J and K in S1 File), the coefficients for perceived invincibility were negative and significant across all ages except those under 20 and over 80. The coefficients for cultural collectivism were negative and significant only for the ages 61 to 70 and 71 to 80. The interaction effects between perceived invincibility and cultural collectivism were significant and positive for all ages except those under 30, 61 to 70, and over 80. See S1 File for all estimated beta coefficients.

We conducted a similar analysis, running separate regressions for both male and female respondents. For prosocial concern (Tables L and M in S1 File), the estimated coefficients for perceived invincibility were negative and significant for males and females (*b* = -.862, *t* = -13.11, *p* < 0.01; *b* = -.625, *t* = -9.30, *p* < 0.01). Using seemingly unrelated regression and testing across the regression models shows the coefficients for perceived invincibility were significantly different for males compared to females (*Chi-square*(1) = 19.44, *p* < 0.01). The estimated coefficients for the interaction between cultural collectivism and perceived invincibility were significant and positive for males and females (*b* = .009, *t* = 39.04, *p* < 0.01; *b* = .007, *t* = 33.23, *p* < 0.01). Testing across the models shows the coefficients for the interaction effects were significantly different for males compared to females (*Chi-square*(1) = 4.73, *p* < 0.05).

For vaccine intention (Tables N and O in S1 File), the estimated coefficients for perceived invincibility were negative and significant for males and females (*b* = -.751, *t* = -24.46, *p* < 0.01; *b* = -.554, *t* = -18.62, *p* < 0.01). Using seemingly unrelated regression and testing across the regression models shows the coefficients for perceived invincibility were significantly different for males compared to females (*Chi-square*(1) = 11.05, *p* < 0.01). The estimated coefficients for the interaction between cultural collectivism and perceived invincibility were significant and positive for males and females (*b* = .007, *t* = 11.88, *p* < 0.01; *b* = .005, *t* = 8.95, *p* < 0.01). Testing across the models shows the coefficients for the interaction effects are not significantly different for males compared to females (*Chi-square*(1) = 1.52, *p* = 0.22).

Overall, the relationship between perceived invincibility and cultural collectivism on prosocial concern and vaccine intention appears to be robust across age cohort and gender. Interestingly, the magnitude of the coefficients did not demonstrate any pattern of decline as age increased. This suggests that despite invincibility being correlated with age, higher perceptions of invincibility were associated with lower prosocial concern and vaccination intention across a wide age range. This may be particularly problematic in the case of COVID-19 as age is a known risk factor in disease severity [34]. Also, while the magnitudes of the effects of perceived invincibility on prosocial concern and vaccination intention were greater for males compared to females, the effects for females were still significant.

## Discussion

Overall, the results provide initial insights on the relationships between perceived invincibility from COVID-19 infection, cultural collectivism, prosocial concern, and vaccination intention. Perceived invincibility from COVID-19 infection was found to have negative effects on prosocial concern (*n* = 218,956) and vaccination intention (*n* = 71,148). Importantly, however, cultural collectivism modulated these relationships such that the magnitude of the effects of perceived invincibility on prosocial concern and vaccination intention were less pronounced among participants in cultures with high cultural collectivism. These results support our hypotheses and provide insight on the interplay between country-level and individual-level factors that could affect the beliefs and behaviors needed to suppress the spread of a highly contagious disease at global scale.

## Conclusions

This research adds to our understanding of the individual- and cultural-level drivers of prosocial beliefs and behaviors during a pandemic. Our results suggest that perceived invincibility from COVID-19 infection may threaten a community’s ability to suppress the disease. Prior research indicates that perceived invincibility threatens an individual’s health and wellness [20, 21, 23–26, 28]. Building on this research, we find that perceived invincibility may also threaten collective health and wellbeing by inhibiting the individual beliefs and actions needed to suppress a highly contagious disease, such as COVID-19. However, we also find that the threat of perceived invincibility varies across cultures.

Building on cultural dimensions theory [48], we find that in collectivistic cultures, which value interdependence and collective wellbeing, perceived invincibility is less threatening to prosocial concern and vaccination intentions. However, in less collectivistic cultures, which value personal freedoms and autonomy, perceived invincibility may threaten community efforts to suppress a pandemic. Prior commentary has alluded to this relationship, noting that perceived invincibility in developed countries, such as the U.S., is a potential threat to achieving the collective preventative behavior necessary to suppress COVID-19 [5, 29]. The present research provides initial empirical evidence of this relationship at global scale.

### Implications for theory and practice

During the current pandemic, vaccination acceptance rates have varied across countries. Recent polling found that most adults (about 70%) across the world are willing to take a vaccine for COVID-19 [63]; however, vaccination acceptance remains low among some populations, possibly threatening our ability to achieve worldwide herd immunity [2, 64]. Thus, it remains critical to identify factors affecting vaccination acceptance. Research has identified demographic and sociodemographic factors as well as the proliferation misinformation which may increase vaccine hesitancy [9, 11, 12, 65]. Political orientation and differential exposure to media outlets and social networks may also affect attitudes toward vaccines [14]. The present research contributes to prior research on vaccine hesitancy by bridging prior work on cultural collectivism [8] and perceived invincibility [19, 20].

Our results suggest that despite the prevalence of personal feelings of invincibility across cultures, the effects of perceived invincibility on prosocial beliefs and behaviors depend on the extent to which individuals maintain an interdependent self-construal and feel responsible for the wellbeing of their community members [37, 46]. In turn, health communications should encourage a collectivistic mindset so as to promote prosocial beliefs and behaviors, despite personal feelings of invincibility. In the context of COVID-19, this is especially important in less collectivistic or individualistic cultures such as the U.S., U.K., and France. To increase COVID-19 vaccination uptake in less collectivistic cultures, we recommend policy and health communications promote feelings of interdependence among community members by highlighting their shared goals, beliefs, and values. This approach was recently evident in U.S. President Joe Biden’s public remarks, “I need the American people to do their part…It’s a patriotic duty. It’s the only way we ever get back to normal—to cheer together in stadiums full of fans; to gather together on holidays again safely; go to graduations, weddings.” [66].

### Limitations and future research

Given the widespread and deleterious effects of perceived invincibility on health-related behaviors, such as reckless driving and sexual activity, binge drinking, and low receptivity to health communications [20, 24, 26, 28], future research is needed that considers the potential modulating role of one’s cultural orientation. As with the current research, such research could better inform policy and health communication efforts in promoting health and wellbeing. In general, perceived invincibility has been an understudied construct in cross-cultural health research, and additional work is needed to further articulate the relationships between perceived invincibility, culture, and health-related beliefs and behaviors.

Future research on perceived invincibility across cultures would benefit from the development and validation of a cross-cultural perceived invincibility scale. The Adolescent Invincibility Test (AIT) has been used to measure perceived invincibility in adolescent populations [20] and the Invincibility Belief Index (IBI) was developed to assess military personnel [18]. However, the ability of these scales to measure perceived invincibility across cultures has not been demonstrated.

Building on the present research, research is also needed to assess cultural collectivism at the individual or regional level, rather than at the country level. Cultural collectivism can vary substantially both within and across countries [8, 58, 59, 67]. Vandello and Cohen [59] provide cultural collectivism scores across the U.S. and, using these scores and other proxy measures for cultural collectivism, recent research has found associations between collectivism and COVID-19 cases and deaths in the U.S. [68, 69]. The mixed results of these studies suggest the need to consider regional differences in collectivism, in addition to country-level differences, when developing policy and heath communications to promote preventative behavior during a pandemic.

## Supporting information

S1 File Supporting material and analysis. (PDF)
