# Supplementary material for: Invincibility threatens vaccination intentions during a pandemic
Source: PLoS One. 2021 Oct 27;16(10):e0258432. doi: 10.1371/journal.pone.0258432 (PMC8550387; doi:10.1371/journal.pone.0258432)
Supplement: S1 File — (PDF) [file pone.0258432.s001.pdf]

S1 File: Supporting Materials and Analysis

Invincibility threatens vaccination intentions during a pandemic

James M. Leonhardt,[1*] Garret Ridinger,[2] Yu Rong,[2] Amir Talaei-Khoe[3]

[1] Department of Marketing, University of Nevada, Reno, Nevada, United States of America

[2] Department of Management, University of Nevada, Reno, Nevada, United States of America

[3] Department of Information Systems, University of Nevada, Reno, Nevada, United States of America

* Corresponding author; email: jleonhardt@unr.edu

The following Supporting Information is available for this article:

Data 1. Individual-level data. Description and link to request dataset.

Data 2. Country-level data. Description and link to access dataset.

Fig A. Individual-level data participant recruitment example.

Table A. Descriptive statistics and correlations for prosocial concern.

Table B. Descriptive statistics and correlations for vaccine intention.

Table C. Country-level cultural collectivism index scores.

Additional analysis 1. Alternative model specifications.

Table D. Ordinary least square results for the effects of perceived invincibility and cultural collectivism on prosocial concern.

Table E. Multinomial logit results for the effects of perceived invincibility and cultural collectivism on prosocial concern.

Table F. Ordinary least square results for the effects of perceived invincibility and cultural collectivism on vaccine intention.

Table G. Multinomial logit results for the effects of perceived invincibility and cultural collectivism on vaccine intention.

Additional analysis 2. Age and gender.

Table H. Ordinary least square results for the effects of perceived invincibility and cultural collectivism on prosocial concern by age cohort (50 and under).

Table I. Ordinary least square results for the effects of perceived invincibility and cultural collectivism on prosocial concern by age cohort (over 50).

Table J. Ordinary least square results for the effects of perceived invincibility and cultural collectivism on vaccine intention by age cohort (50 and under).

Table K. Ordinary least square results for the effects of perceived invincibility and cultural collectivism on vaccine intention by age cohort (over 50).

Table L. Ordinary least square results for the effects of perceived invincibility and cultural collectivism on prosocial concern by sex (male).

Table M. Ordinary least square results for the effects of perceived invincibility and cultural collectivism on prosocial concern by sex (female).

Table N. Ordinary least square results for the effects of perceived invincibility and cultural collectivism on vaccine intention by sex (male).

Table O. Ordinary least square results for the effects of perceived invincibility and cultural collectivism on vaccine intention by sex (female).

S1 File References. References included in the S1 File.

Data 1. Individual-level data

Individual-level data was sourced from the Beliefs, Behaviors, and Norms Survey [1,2]. Collis et al. [2] provide details on the motivation behind the design of the survey and the sampling methodology used to ensure representative samples from each country. The survey dataset (deidentified) is available to academics and nonprofits upon completion of the Facebook Data Use Agreement at https://dataforgood.fb.com/docs/preventive-health-survey-request-for-data-access/. Fig A in S1 provides an example of the display ads used on Facebook to recruit BBNS participants.

From the BBNS, we sourced the following measures to assess our hypotheses: Perceived invincibility to COVID-19 infection corresponds to the following BBNS measure: (String Variable) How serious would it be if you became infected with COVID-19? [Not at all serious, Somewhat serious, Very serious]. Prosocial concern about taking actions to prevent the spread of COVID-19 corresponds to the following BBNS measure: (String Variable) How important is it for you to take actions to prevent the spread of COVID-19 in your community? [Extremely important, Very important, Moderately important, Slightly important, Not important at all]. Vaccination intention corresponds to the following BBNS measure: (String Variable) If a vaccine against COVID-19 infection is available in the market, would you take it? [Yes, definitely; Probably; Unsure; Probably not; No, definitely not].

Several control variables were also collected. Perceived personal health corresponds to the following BBNS measure: (String Variable) In general, how would you rate your overall health? [Excellent, Very good, Good, Fair, Poor]. Participant's age corresponds to the following BBNS measure: (String Variable) [Under 20, 20-30, 31-40, 41-50, 51-60, 61-70, 71-80, Over 80]. Participant's sex corresponds to the following BBNS measure: (String Variable) [Male,

Female, Other]. Participant's education corresponds to the following BBNS measure: (String Variable) What is the highest level of education you have completed? [Less than primary school, Primary school, Secondary school, College/university, Graduate school]. Participant's country was assessed using metadata including the geographic location of the IP address of the survey participant as well as participant's self-reported country: (String Variable) In which country do you currently reside? In cases of missing self-reported country, the geographical IP address was used to infer country. See Tables A and B for descriptive statistics and correlations between these variables.

Data 2. Country-level data

The country-level dataset is publicly available from the OSF public repository at https://osf.io/qwn9f/?view_only=4349d3e12ade40b99b10053cf4f8fdf8. Country-level data on cultural collectivism was sourced from Fincher et al. [3] and Webster et al. [4]. The data includes country-level collectivism scores from four prior studies, including Hofstede [5], Suh et al. [6], Gelfand et al. [7], and Kashima and Kashima [8]. The Hofstede [5] data consists of individualism-collectivism scores from surveys conducted on over 100,000 worldwide IBM employees. The Hofstede [5] data provided by Webster [4] was compiled from Fincher et al. [3] and Geert Hofstede's public website [9]. The Suh et al. [6] data consists of individualism-collectivism scores for countries based on estimates from Hofstede's [7] and estimates from Triandis [10]. The Gelfand et al. [9] data consists of collectivism scores based on 17,370 responses to their Global Leadership and Organizational Behavior Effectiveness (GLOBE) Research Program. The Kashima and Kashima [8] data consists of a binary measure of collectivism across countries based on the spoken language of the country and the acceptability of omitting first (e.g., "I") and second (e.g., "you") pronouns.

The country-level data was matched with 51 countries for which there were corresponding individual-level data from the BBNS. For each of the 51 countries (see Table C), a composite collectivism score was created following Fincher et al. [3] and Webster et al. [4]. For the syntax and the corresponding output for this transformation see S1 Syntax and S1 Output, respectively. First, each of the four country-level scores were standardized using z-scores. Second, the Hofstede [5] and the Suh et al. [6] scores were reverse scored (z * -1) so that higher scores would indicate higher collectivism to align with the other two scores. Bivariate correlations between these four scores were then assessed, and the mean of the four scores was taken to create a composite measure. Finally, the composite measure was linearly transformed to create a Cultural Collectivism Index score with a mean of about 50 and a SD of about 20 (see Table C) to aide in the interpretability of our findings relative to prior cross-cultural research on collectivism [3-5].

Fig A. Example of Participant Recruitment for the BBNS.

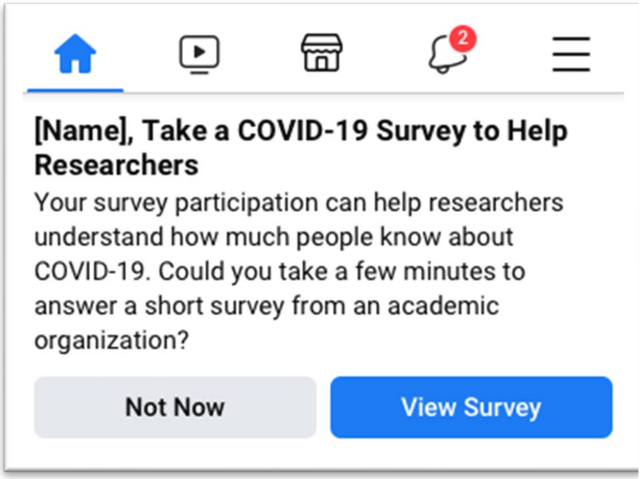

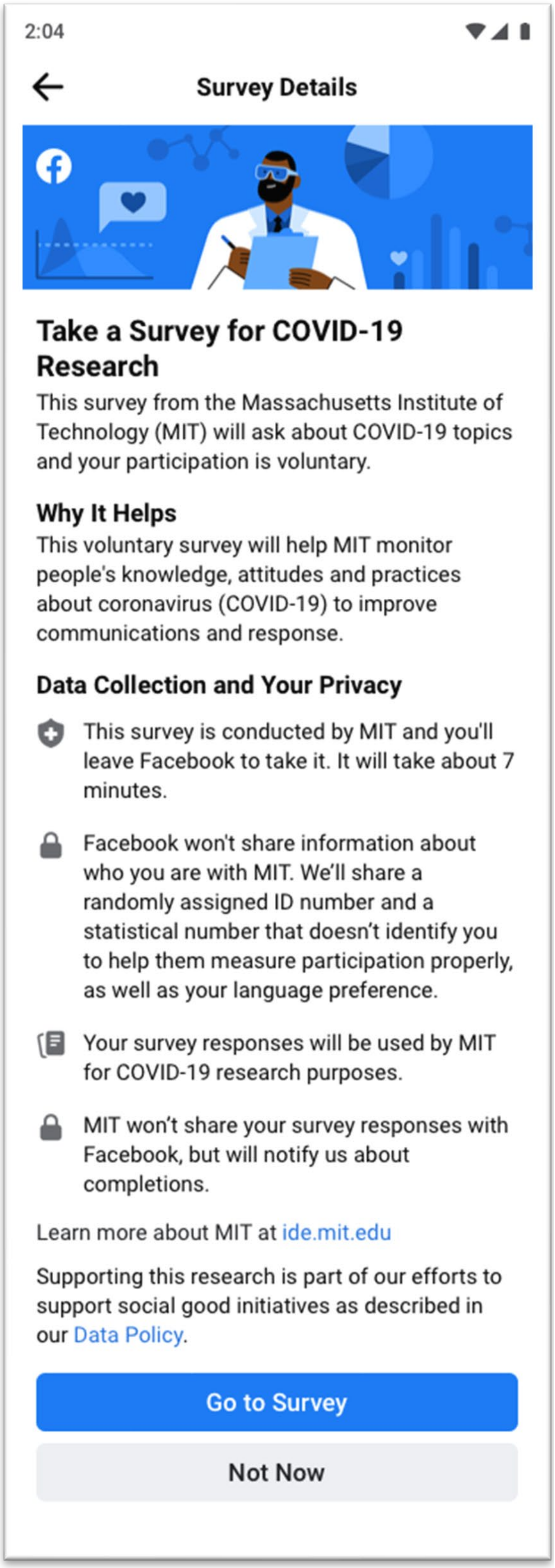

Table A. Descriptive Statistics and Correlations for Prosocial Concern

		Mean	SD	1	2	3	4	5	6	7
1	Sex	0.54	0.50	1						
2	Age	3.59	1.50	0.02	1					
3	Education	3.81	0.79	0.02	-0.05	1				
4	Collectivism	50.37	17.44	0.15	-0.26	0.03	1			
5	Own Health	3.42	0.97	0.07	-0.21	0.11	0.03	1		
6	Perceived Invincibility	1.69	0.68	0.02	-0.08	0.04	-0.10	0.17	1	
7	Prosocial Concern	4.34	0.86	-0.09	0.02	0.05	0.05	0.03	-0.28	1

Note: # of Observations = 218,956

Table B. Descriptive Statistics and Correlations for Vaccine Intention

		Mean	SD	1	2	3	4	5	6	7
1	Sex	0.54	0.50	1						
2	Age	3.63	1.52	0.02	1					
3	Education	3.81	0.78	0.02	0.04	1				
4	Collectivism	50.65	17.64	0.13	-0.25	0.04	1			
5	Own Health	3.39	0.98	0.08	-0.19	0.11	0.02	1		
6	Perceived Invincibility	1.70	0.68	0.03	-0.10	0.04	-0.07	0.18	1	
7	Vaccine Intention	3.86	1.27	0.10	0.01	0.06	0.06	-0.04	-0.19	1

Note: # of Observations = 71,148

Table C. Country-level Cultural Collectivism Index Scores

Country	ID	Cultural Collectivism Index
Argentina	1	52.81
Australia	2	13.06
Bangladesh	3	64.95
Bolivia	4	54.60
Brazil	5	54.10
Canada	6	16.88
Chile	7	60.02
Colombia	8	67.16
Ecuador	9	66.79
Egypt	10	58.26
Estonia	11	45.12
France	12	24.93
Georgia	13	75.49
Germany	14	20.41
Ghana	15	49.01
Guatemala	16	65.59
India	17	56.30
Indonesia	18	66.48
Iraq	19	61.22
Italy	20	38.02
Jamaica	21	49.56
Japan	22	47.57
Kazakhstan	23	48.51
Kenya	24	60.25
Malaysia	25	59.02
Mexico	26	59.33
Morocco	27	62.30
Nepal	28	66.86
Netherlands	29	12.82
Nigeria	30	50.99
Pakistan	31	68.41
Peru	32	66.04
Philippines	33	65.63
Poland	34	49.59
Portugal	35	58.83
Romania	36	55.54
Singapore	37	62.00
South Africa	38	28.46
South Korea	39	64.20
Spain	40	49.65
Taiwan	41	61.44
Tanzania	42	60.25
Thailand	43	63.58
Trinidad	44	68.19
Turkey	45	59.49
United Arab Emirates	46	61.22
United Kingdom	47	12.72
United States	48	12.18
Uruguay	49	56.60
Venezuela	50	63.00
Vietnam	51	64.95
Mean (SD)		52.56 (16.87)

Note: The country-level dataset is publicly available from the OSF public repository at https://osf.io/qwn9f/?view_only=4349d3e12ade40b99b10053cf4f8fdf8.

Additional analysis 1. Alternative model specifications

To test the robustness of the results presented in the main paper we conducted additional model specifications. Table D in S1 repeats the analysis from Table 1 in the main paper but uses Ordinary Least Squares with clustered standard errors at the country level. Since the variable prosocial concern takes on six possible values, we also conducted multinomial logit regressions treating prosocial concern as a categorical variable. The coefficients, reported in Table D in S1, are relative risk ratios (RRR). Estimates for each column are relative to selecting "Not important at all" for prosocial concern. Both Tables D and E in S1 show that the results are consistent with those in Table 1 in the main paper.

Table D. Ordinary Least Square Results for the Effects of Perceived Invincibility and Cultural Collectivism on Prosocial Concern.

	(1)					(2)				
	**Est.**	**SE**	**p-Value**	**95% CI**		**Est.**	**SE**	**p-Value**	**95% CI**	
				LL	***UL***				***LL***	***UL***
Constant	5.05	0.11	<0.001	4.82	5.28	4.18	0.11	<0.001	3.97	4.40
Perceived Invincibility (PI)	-0.73	0.06	<0.001	-0.86	-0.61					
Cultural Collectivism (CC)	-0.01	0.00	<0.001	-0.01	-0.01	-0.00	0.00	0.234	-0.00	0.00
PI x CC	0.01	0.00	<0.001	0.01	0.01					
High Perceived Invincibility (High PI)						-1.69	0.14	<0.001	-1.97	-1.41
Medium Perceived Invincibility (Medium PI)						-0.41	0.04	<0.001	-0.49	-0.32
High PI x CC						0.02	0.00	<0.001	0.01	0.02
Medium PI x CC						0.00	0.00	<0.001	0.00	0.00
Age	0.01	0.01	0.170	-0.01	0.03	0.01	0.01	0.162	-0.01	0.03
Sex	-0.15	0.02	<0.001	-0.19	-0.12	-0.15	0.02	<0.001	-0.19	-0.12
Education	0.06	0.02	<0.001	0.03	0.10	0.06	0.02	<0.001	0.02	0.09
Health	0.08	0.01	<0.001	0.05	0.10	0.08	0.01	<0.001	0.06	0.10
R-squared	0.11					0.11				
N	218,956					218,956				

Note: Standard errors are clustered at the country level. Additional control variables include date survey was completed.

Table E. Multinomial Logit Results for the Effects of Perceived Invincibility and Cultural Collectivism on Prosocial Concern.

	(2) Slightly Important		(3) Moderately Important		(4) Very Important		(5) Extremely Important	
	RRR (SE)	95% CI	RRR (SE)	95% CI	RRR (SE)	95% CI	RRR (SE)	95% CI
Constant	35.94** (19.32)	[12.1,103]	1487** (1309)	[0.26,0.54]	8002** (6820)	[1505, 42526]	26302** (23829)	[4455, 155292]
Perceived Invincibility (PI)	0.37** (0.07)	[0.26,0.54]	0.14** (0.03)	[0.09,0.22]	0.05** (0.01)	[0.03,0.08]	0.02** (0.01)	[0.01,0.03]
Cultural Collectivism (CC)	0.99 (0.01)	[0.97,1.00]	1.01** (0.01)	[1.00,1.03]	0.95** (0.01)	[0.93,0.98]	0.94** (0.01)	[0.91,0.96]
PI x CC	1.00 (0.00)	[1.00,1.01]	1.02** (0.01)	[1.01,1.03]	1.03** (0.01)	[1.02,1.04]	1.03** (0.01)	[1.02,1.05]
Age	1.00 (0.04)	[0.93,1.08]	1.05 (0.09)	[0.89,1.24]	1.12* (0.05)	[1.03,1.23]	1.10* (0.05)	[1.01,1.20]
Sex	0.75** (0.06)	[0.65,0.87]	0.61** (0.04)	[0.53,0.70]	0.48** (0.04)	[0.41,0.57]	0.38** (0.04)	[0.31,0.47]
Education	1.12* (0.05)	[1.02,1.23]	1.14* (0.07)	[1.01,1.29]	1.27* (0.11)	[1.07,1.49]	1.40* (0.13)	[1.16,1.68]
Health	0.78** (0.03)	[0.72,0.85]	0.71** (0.06)	[0.61,0.83]	0.86** (0.04)	[0.78,0.95]	1.05 (0.06)	[0.95,1.17]
Pseudo R-squared	0.05							
N	218,956							

Note: Standard errors in parentheses are clustered at the country level. Estimates for each column are relative to selecting "Not important at all" for prosocial concern. Reported coefficients are Relative Risk Ratios (RRR). Additional control variables include date survey was completed. **p*<0.05, ***p*<0.01

Similarly, Table F in S1 repeats the analysis from Table 2 in the main paper but uses Ordinary Least Squares with clustered standard errors at the country level. Table G reports multinomial logit regressions treating vaccine intention as a categorical variable. Estimates for each column are relative to selecting "No, definitely not" for vaccine intention. Both Tables F and G in S1 show that the results are consistent with those in Table 2 in the main paper.

Table F. Ordinary Least Square Results for the Effects of Perceived Invincibility and Cultural Collectivism on Vaccine Intention.

	(1)					(2)				
	**Est.**	**SE**	**p-Value**	**95% CI**		**Est.**	**SE**	**p-Value**	**95% CI**	
				LL	***UL***				***LL***	***UL***
Constant	4.33	0.15	<0.001	4.04	4.63	3.55	0.15	<0.001	3.26	3.85
Perceived Invincibility (PI)	-0.62	0.06	<0.001	-0.76	-0.49					
Cultural Collectivism (CC)	-0.01	0.00	0.023	-0.01	-0.00	0.00	0.00	0.837	-0.00	0.01
PI x CC	0.01	0.00	<0.001	0.00	0.01					
High Perceived Invincibility (High PI)						-1.49	0.14	<0.001	-1.77	-1.21
Medium Perceived Invincibility (Medium PI)						-0.24	0.07	<0.001	-0.38	-0.10
High PI x CC						0.01	0.00	<0.001	0.01	0.02
Medium PI x CC						0.00	0.00	0.465	-0.00	0.00
Age	-0.01	0.00	0.487	-0.03	0.01	-0.01	0.01	0.523	-0.03	0.01
Sex	0.26	0.03	<0.001	0.19	0.33	0.27	0.03	<0.001	0.19	0.34
Education	0.12	0.03	<0.001	0.05	0.18	0.11	0.03	<0.001	0.05	0.17
Health	-0.02	0.01	0.048	-0.05	-0.00	-0.02	0.01	0.124	-0.04	0.17
R-squared	0.06					0.07				
N	71,148					71,148				

Note: Standard errors are clustered at the country level. Additional control variables include date survey was completed.

Table G. Multinomial Logit Results for the Effects of Perceived Invincibility and Cultural Collectivism on Vaccine Intention

	(2) Probably Not		(3) Unsure		(4) Probably		(5) Yes, definitely	
	RRR (SE)	95% CI	RRR (SE)	95% CI	RRR (SE)	95% CI	RRR (SE)	95% CI
Constant	5.08** (1.82)	[2.51,10.27]	31.18** (12.66)	[14.1,69.1]	23.51** (11.71)	[1505, 42526]	41.43** (21.37)	[15.1, 113]
Perceived Invincibility (PI)	0.47** (0.05)	[0.38,0.57]	0.30** (0.05)	[0.22,0.40]	0.30** (0.06)	[0.21,0.44]	0.19** (0.03)	[0.14,0.27]
Cultural Collectivism (CC)	0.98** (0.01)	[0.97,1.00]	0.98* (0.01)	[0.97,1.00]	0.98 (0.01)	[0.96,1.00]	0.98 (0.01)	[0.96,1.00]
PI x CC	1.01** (0.00)	[1.00,1.02]	1.01** (0.00)	[1.01,1.02]	1.01** (0.00)	[1.00,1.02]	1.01** (0.00)	[1.01,1.02]
Age	0.96 (0.02)	[0.92,1.00]	0.98 (0.02)	[0.93,1.02]	0.93* (0.03)	[0.87,0.99]	0.97 (0.03)	[0.92,1.02]
Sex	0.99 (0.05)	[0.90,1.09]	0.92 (0.06)	[0.81,1.05]	1.32** (0.10)	[1.14,1.52]	1.65** (0.15)	[1.38,1.97]
Education	1.23** (0.08)	[1.08,1.39]	1.15 (0.09)	[0.99,1.35]	1.30** (0.12)	[1.09,1.55]	1.40** (0.13)	[1.16,1.68]
Health	0.81** (0.02)	[0.76,0.85]	0.77** (0.03)	[0.72,0.83]	0.74** (0.03)	[0.68,0.80]	0.83** (0.03)	[0.77,0.90]
Pseudo R-squared	0.03							
N	71,148							

Note: Standard errors in parentheses are clustered at the country level. Estimates for each column are relative to selecting “No, definitely not” for vaccine intention. Reported coefficients are Relative Risk Ratios (RRR). Additional control variables include date survey was completed. *$p < 0.05$, **$p < 0.01$

Additional analysis 2. Age and gender

To test the robustness of the results across age cohorts, we repeated our analysis from Table F using Ordinary Least Squares with clustered standard errors at the country level restricted the sample by age cohort. We note that alternative specifications using multi-level modeling using the approach in tables 1 and 2 in the main paper give similar results. Specifically, we conducted regressions for each age group: under 20, 21 to 30, 31 to 40, 41 to 50, 61 to 70 and over 80. Tables H and I contain the results for predicting preventative behaviors for each age cohort. Tables J and K present the results predicting vaccine intention for each age cohort.

To test the robustness of the results for female and male respondents, we repeated the analysis from Table F using Ordinary Least Squares with clustered standard errors at the country level. We note that alternative specifications using multi-level modeling like Tables 1 and 2 in the main paper give similar results. Specifically, we conducted regressions restricted data to include only male and only female respondents. Tables L and M contain results for predicting preventative behaviors for males and females. Tables N and O contain results for predicting vaccine intention for males and females.

Table H. Ordinary Least Square Results for the Effects of Perceived Invincibility and Cultural Collectivism on Prosocial Concern by Age Cohort (50 and under)

	(1) Under 20		(2) 20 to 30		(3) 31 to 40		(4) 41 to 50	
	Est (SE)	95% CI	Est (SE)	95% CI	Est (SE)	95% CI	Est (SE)	95% CI
Constant	4.48** (0.31)	[3.86,5.09]	4.87** (0.17)	[4.52,5.22]	5.13** (0.13)	[2.92,6.07]	5.25** (0.12)	[5.01, 5.50]
Perceived Invincibility (PI)	-0.51** (0.11)	[-0.73,-0.30]	-0.64** (0.08)	[-0.80,-0.48]	-0.76** (0.07)	[-0.89,-0.63]	-0.80** (0.07)	[-0.94,-0.66]
Cultural Collectivism (CC)	0.00 (0.00)	[0.00,0.01]	-0.01* (0.00)	[-0.01,-0.00]	-0.01** (0.00)	[-0.02,-0.01]	-0.01** (0.00)	[-0.02,-0.01]
PI x CC	0.00* (0.00)	[0.00,0.01]	0.01** (0.00)	[0.00,0.01]	0.01** (0.00)	[0.01,0.01]	0.01** (0.00)	[0.01,0.01]
Sex	-0.14** (0.03)	[-0.21,-0.08]	-0.15** (0.02)	[-0.19,-0.11]	-0.15** (0.02)	[-0.19,-0.11]	-0.14** (0.02)	[-0.18,-0.10]
Education	0.08 (0.05)	[-0.03,0.18]	0.08* (0.03)	[0.02,0.14]	0.07** (0.02)	[0.04,0.11]	0.07** (0.02)	[0.03,0.10]
Health	0.05* (0.02)	[0.01,0.08]	0.06** (0.01)	[0.04,0.09]	0.08** (0.01)	[0.05,0.11]	0.07** (0.01)	[0.05,0.10]
R-squared	0.08		0.09		0.11		0.11	
N	7,388		55,584		52,649		41,912	

Note: Standard errors in parentheses are clustered at the country level. Estimates for each column are restricted by age cohort. Additional control variables include date survey was completed. **p* < 0.05, ***p* < 0.01

Table I. Ordinary Least Square Results for the Effects of Perceived Invincibility and Cultural Collectivism on Prosocial Concern by Age Cohort (Over 50)

	**(1)**		**(2)**		**(3)**		**(4)**	
	**51 to 60**		**61 to 70**		**71 to 80**		**Over 80**	
	Est (SE)	95% CI	Est (SE)	95% CI	Est (SE)	95% CI	Est (SE)	95% CI
Constant	5.30** (0.11)	[5.08,5.53]	5.19** (0.10)	[5.00,5.39]	5.11** (0.11)	[4.90,5.33]	5.18** (0.28)	[4.73, 5.64]
Perceived Invincibility (PI)	-0.79** (0.07)	[-0.94,-0.64]	-0.72** (0.06)	[-0.85,-0.60]	-0.67** (0.06)	[-0.79,-0.55]	-0.85** (0.12)	[-1.09,-0.60]
Cultural Collectivism (CC)	-0.01** (0.00)	[-0.01,-0.00]	-0.01** (0.00)	[-0.01,-0.01]	-0.01** (0.00)	[-0.01,-0.00]	-0.01 (0.00)	[-0.02,0.00]
PI x CC	0.01** (0.00)	[0.00,0.01]	0.01** (0.00)	[0.00,0.01]	0.01** (0.00)	[0.00,0.01]	0.01 (0.00)	[-0.00,0.01]
Sex	-0.17** (0.02)	[-0.21,-0.13]	-0.18** (0.03)	[-0.24,-0.13]	-0.23** (0.03)	[-0.28,-0.17]	-0.20* (0.08)	[-0.35,-0.04]
Education	0.04** (0.01)	[0.01,0.06]	0.03* (0.01)	[0.00,0.06]	0.02 (0.02)	[-0.02,0.06]	0.03 (0.03)	[--0.04,0.10]
Health	0.09** (0.01)	[0.06,0.11]	0.10** (0.02)	[0.06,0.13]	0.09** (0.02)	[0.06,0.13]	0.12** (0.00)	[0.04,0.19]
R-squared	0.12		0.13		0.14		0.18	
N	34,186		19,908		6,399		930	

Note: Standard errors in parentheses are clustered at the country level. Estimates for each column are restricted by age cohort. Additional control variables include date survey was completed. *$p < 0.05$, **$p < 0.01$

Table J. Ordinary Least Square Results for the Effects of Perceived Invincibility and Cultural Collectivism on Vaccine Intention by Age Cohort (50 and under)

	(1) Under 20		(2) 20 to 30		(3) 31 to 40		(4) 41 to 50	
	Est (SE)	95% CI	Est (SE)	95% CI	Est (SE)	95% CI	Est (SE)	95% CI
Constant	5.03** (0.75)	[3.43,6.63]	4.27** (0.58)	[3.10,5.44]	4.50** (0.79)	[2.92,6.07]	4.49** (0.71)	[3.06, 5.92]
Perceived Invincibility (PI)	-0.15 (0.13)	[-0.41,0.02]	-0.41** (0.11)	[0.22,0.40]	-0.57** (0.11)	[-0.80,-0.35]	-0.63** (0.08)	[-0.80,-0.47]
Cultural Collectivism (CC)	0.01 (0.01)	[-0.00,0.02]	0.00 (0.00)	[-0.00,0.01]	-0.00 (0.00)	[-0.01,0.01]	-0.01 (0.00)	[-0.01,0.00]
PI x CC	-0.00 (0.00)	[-0.01,0.00]	0.00 (0.00)	[-0.00,0.01]	0.00* (0.00)	[0.00,0.01]	0.01** (0.00)	[0.00,0.01]
Sex	0.12 (0.07)	[-0.02,0.26]	0.16** (0.04)	[0.07,0.25]	0.25** (0.04)	[0.16,0.33]	0.31** (0.04)	[0.23,0.39]
Education	0.11* (0.05)	[0.00,0.21]	0.14** (0.03)	[0.07,0.20]	0.17** (0.05)	[0.07,0.26]	0.12** (0.05)	[0.03,0.21]
Health	-0.06* (0.02)	[-0.11,-0.00]	-0.04** (0.01)	[-0.06,-0.01]	-0.01 (0.02)	[-0.04,0.02]	-0.03* (0.01)	[-0.06,-0.00]
R-squared	0.04		0.06		0.06		0.06	
N	2,456		17,502		16,822		13,508	

Note: Standard errors in parentheses are clustered at the country level. Estimates for each column are restricted by age cohort. Additional control variables include date survey was completed. **p* < 0.05, ***p* < 0.01

Table K. Ordinary Least Square Results for the Effects of Perceived Invincibility and Cultural Collectivism on Vaccine Intention by Age Cohort (Over 50)

	(1) 51 to 60		(2) 61 to 70		(3) 71 to 80		(4) Over 80	
	Est (SE)	95% CI	Est (SE)	95% CI	Est (SE)	95% CI	Est (SE)	95% CI
Constant	4.64** (0.59)	[3.46,5.82]	4.27** (0.58)	[3.10,5.44]	4.28** (0.60)	[3.07,5.49]	3.94** (1.22)	[1.49, 6.40]
Perceived Invincibility (PI)	-0.62** (0.08)	[-0.77,-0.46]	-0.51** (0.08)	[-0.67,-0.36]	-0.58** (0.06)	[-0.69,-0.46]	-0.21 (0.13)	[-0.46,0.05]
Cultural Collectivism (CC)	-0.01 (0.00)	[-0.01,0.00]	-0.01* (0.00)	[-0.00,0.01]	-0.01** (0.00)	[-0.02,-0.01]	-0.00 (0.00)	[-0.01,0.00]
PI x CC	0.00* (0.00)	[0.00,0.01]	0.00 (0.00)	[-0.00,0.01]	0.00* (0.00)	[0.00,0.01]	-0.01 (0.00)	[-0.01,0.00]
Sex	0.33** (0.04)	[0.25,0.42]	0.32** (0.04)	[0.24,0.41]	0.36** (0.04)	[0.27,0.45]	0.58** (0.18)	[0.20,0.96]
Education	0.09** (0.03)	[0.03,0.15]	0.07* (0.03)	[0.01,0.13]	0.02 (0.03)	[-0.04,0.09]	0.08 (0.09)	[-0.10,0.02]
Health	-0.03 (0.02)	[-0.07,0.00]	0.01 (0.02)	[-0.03,0.05]	0.04 (0.03)	[-0.03,0.10]	0.02 (0.08)	[-0.14,0.18]
R-squared	0.07		0.06		0.07		0.12	
N	11,455		6,732		2,323		360	

Note: Standard errors in parentheses are clustered at the country level. Estimates for each column are restricted by age cohort. Additional control variables include date survey was completed. *$p < 0.05$, **$p < 0.01$

Table L. Ordinary Least Square Results for the Effects of Perceived Invincibility and Cultural Collectivism on Prosocial Concern by Sex (Male).

	(1)					(2)				
	Est.	SE	p-Value	95% CI		Est.	SE	p-Value	95% CI	
				LL	*UL*				*LL*	*UL*
Constant	5.14	0.14	<0.001	4.86	5.42	4.11	0.12	<0.001	3.87	4.34
Perceived Invincibility (PI)	-0.86	0.07	<0.001	-0.99	-0.73					
Cultural Collectivism (CC)	-0.01	0.00	<0.001	-0.02	-0.01	-0.00	0.00	0.337	-0.01	0.00
PI x CC	0.01	0.00	<0.001	0.01	0.01					
High Perceived Invincibility (High PI)						-1.88	0.13	<0.001	-2.16	-1.61
Medium Perceived Invincibility (Medium PI)						-0.46	0.05	<0.001	-0.56	-0.35
High PI x CC						0.02	0.00	<0.001	0.01	0.02
Medium PI x CC						0.00	0.00	<0.001	0.00	0.00
Age	0.01	0.01	0.447	-0.01	0.02	0.01	0.01	0.434	-0.01	0.03
Education	0.06	0.02	<0.001	0.02	0.10	0.05	0.02	<0.001	0.02	0.09
Health	0.09	0.01	<0.001	0.06	0.11	0.09	0.01	<0.001	0.07	0.11
R-squared	0.11					0.12				
N	118,376					118,376				

Note: Standard errors are clustered at the country level. Additional control variables include date survey was completed.

Table M. Ordinary Least Square Results for the Effects of Perceived Invincibility and Cultural Collectivism on Prosocial Concern by Sex (Female).

	(1)					(2)				
	**Est.**	**SE**	**p-Value**	**95% CI**		**Est.**	**SE**	**p-Value**	**95% CI**	
				LL	***UL***				***LL***	***UL***
Constant	4.91	0.12	<0.001	4.67	5.15	4.19	0.11	<0.001	3.97	4.41
Perceived Invincibility (PI)	-0.62	0.07	<0.001	-0.76	-0.49					
Cultural Collectivism (CC)	-0.01	0.00	<0.001	-0.01	-0.01	-0.00	0.00	0.337	-0.00	0.00
PI x CC	0.01	0.00	<0.001	0.00	0.01					
High Perceived Invincibility (High PI)						-1.50	0.16	<0.001	-1.83	-1.18
Medium Perceived Invincibility (Medium PI)						-0.37	0.04	<0.001	-0.46	-0.28
High PI x CC						0.02	0.00	<0.001	0.01	0.02
Medium PI x CC						0.00	0.00	<0.001	0.00	0.00
Age	0.02	0.01	0.031	0.00	0.04	0.02	0.01	0.029	0.00	0.04
Education	0.07	0.02	<0.001	0.03	0.10	0.06	0.02	<0.001	0.03	0.10
Health	0.06	0.01	<0.001	0.04	0.08	0.06	0.01	<0.001	0.04	0.08
R-squared	0.08					0.12				
N	100,580					118,376				

Note: Standard errors are clustered at the country level. Additional control variables include date survey was completed.

Table N. Ordinary Least Square Results for the Effects of Perceived Invincibility and Cultural Collectivism on Vaccine Intention by Sex (Male).

	(1)					(2)				
	**Est.**	**SE**	**p-Value**	**95% CI**		**Est.**	**SE**	**p-Value**	**95% CI**	
				LL	***UL***				***LL***	***UL***
Constant	4.87	0.62	<0.001	3.63	6.11	3.91	0.62	<0.001	2.67	5.15
Perceived Invincibility (PI)	-0.75	0.07	<0.001	-0.90	-0.60					
Cultural Collectivism (CC)	-0.01	0.00	<0.001	-0.02	-0.01	-0.00	0.00	0.909	-0.00	0.00
PI x CC	0.01	0.00	<0.001	0.00	0.01					
High Perceived Invincibility (High PI)						-1.71	0.15	<0.001	-2.00	-1.41
Medium Perceived Invincibility (Medium PI)						-0.20	0.07	<0.001	-0.35	-0.05
High PI x CC						0.02	0.00	<0.001	0.01	0.02
Medium PI x CC						-0.00	0.00	0.868	-0.00	0.00
Age	0.00	0.01	0.662	-0.01	0.02	0.00	0.01	0.654	-0.01	0.02
Education	0.13	0.04	<0.001	0.05	0.22	0.13	0.04	<0.001	0.05	0.21
Health	-0.03	0.01	0.012	-0.05	-0.01	-0.02	0.01	0.048	-0.05	-0.00
R-squared	0.06					0.12				
N	38,082					118,376				

Note: Standard errors are clustered at the country level. Additional control variables include date survey was completed.

Table O. Ordinary Least Square Results for the Effects of Perceived Invincibility and Cultural Collectivism on Vaccine Intention by Sex (Female).

	(1)					(2)				
	**Est.**	**SE**	**p-Value**	**95% CI**		**Est.**	**SE**	**p-Value**	**95% CI**	
				LL	***UL***				***LL***	***UL***
Constant	5.07	0.64	<0.001	3.77	6.36	4.39	0.64	<0.001	3.10	5.69
Perceived Invincibility (PI)	-0.55	0.07	<0.001	-0.70	-0.41					
Cultural Collectivism (CC)	-0.01	0.00	0.070	-0.01	0.00	0.00	0.00	0.832	-0.00	0.01
PI x CC	0.01	0.00	<0.001	0.00	0.01					
High Perceived Invincibility (High PI)						-1.33	0.15	<0.001	-1.63	-1.02
Medium Perceived Invincibility (Medium PI)						-0.30	0.08	<0.001	-0.46	-0.13
High PI x CC						0.01	0.00	<0.001	0.01	0.02
Medium PI x CC						0.00	0.00	0.090	-0.00	0.01
Age	-0.02	0.01	0.165	-0.05	0.01	-0.02	0.01	0.189	-0.05	0.01
Education	0.09	0.03	<0.001	0.04	0.15	0.09	0.03	<0.001	0.03	0.14
Health	-0.02	0.02	0.294	-0.05	0.01	-0.01	0.01	0.438	-0.04	0.02
R-squared	0.03					0.04				
N	33,066					33,066				

Note: Standard errors are clustered at the country level. Additional control variables include date survey was completed.

S1 File References.

1. Babalola S, Krenn S, Rimal R, Serlemitsos E, Shaivitz M, Shattuck D, et.al. KAP COVID Dashboard. 2020; Available from: https://ccp.jhu.edu/kap-covid/.
2. Collis A, Garimella K, Moehring A, Rahimian MA, Babalola S, Gobat N, et al. Global survey on COVID-19 beliefs, behaviors, and norms. OSF Preprints; 2021. doi:10.31219/osf.io/7r5sj.
3. Fincher CL, Thornhill R, Murray DR, Schaller M. Pathogen prevalence predicts human cross-cultural variability in individualism/collectivism. Proc Biol Sci. 2008 Jun 7;275(1640):1279-85. doi: 10.1098/rspb.2008.0094
4. Webster GD, Howell JL, Losee JE, Mahar EA, Wongsomboon V. Culture, COVID-19, and collectivism: A paradox of American exceptionalism? Pers Individ Dif. 2021 Aug 1;178:110853.
5. Hofstede G. Culture's consequences: comparing values, behaviors, institutions and organizations across nations. 2nd ed. Thousand Oaks: Sage Publications; 2001 Apr 20.
6. Suh E, Diener E, Oishi S, Triandis HC. The shifting basis of life satisfaction judgments across cultures: emotions versus norms. J Pers Soc Psychol. 1998 Feb;74(2):482-93.
7. Gelfand MJ, Bhawuk DPS, Nishii LH, Bechtold DJ. Individualism and collectivism. In: House RJ, Hanges PJ, Javidan M, Dorfman PW, Gupta V. Culture, leadership, and organizations: the GLOBE study of 62 societies. Thousand Oaks: Sage Publications; 2004. pp. 437–512.
8. Kashima ES, Kashima Y. Culture and language: the case of cultural dimensions and personal pronoun use. J Cross Cult Psychol. 1998 May;29(3):461-86. doi:10.1177/0022022198293005

9. Clearly Cultural: Making Sense of Cross Cultural Communication. 2009 June 19 [cited 2020 Dec 12]. In: Geert Hofstede cultural dimensions [Internet]. Available from: http://clearlycultural.com/geert-hofstede-cultural-dimensions/individualism.
10. Triandis HC. Individualism and collectivism. Boulder: Westview Press; 1995.
